# Estimates of female genital mutilation/cutting in the Netherlands: a comparison between a nationwide survey in midwifery practices and extrapolation-model

**DOI:** 10.1186/s12889-020-09151-0

**Published:** 2020-06-29

**Authors:** Ramin Kawous, Maria E. T. C. van den Muijsenbergh, Diana Geraci, Kyra R. M. Hendriks, Livia E. Ortensi, Femke Hilverda, Alex Burdorf

**Affiliations:** 1 Department of Public Health, Erasmus University Medical Centre, Rotterdam, the Netherlands; 2 Department of Primary and Community Care, Radboud University Medical Centre, Nijmegen, the Netherlands; 3 Pharos, Dutch Centre of Expertise on Health Disparities, Utrecht, the Netherlands; 4 Department of Statistical Sciences “Paolo Fortunati”, Alma Mater Studiorum - University of Bologna, Bologna, Italy; 5 Department of Socio-Medical Sciences, Erasmus School of Health Policy and Management, Erasmus University Rotterdam, Rotterdam, the Netherlands

**Keywords:** Female genital mutilation/cutting, Female circumcision, Prevalence, Direct estimation, Indirect estimation, Midwifery practices, Delivery

## Abstract

**Background:**

Owing to migration, female genital mutilation or cutting (FGM/C) has become a growing concern in host countries in which FGM/C is not familiar. There is a need for reliable estimates of FGM/C prevalence to inform medical and public health policy. We aimed to advance methodology for estimating the prevalence of FGM/C in diaspora by determining the prevalence of FGM/C among women giving birth in the Netherlands.

**Methods:**

Two methods were applied to estimate the prevalence of FGM/C in women giving birth: (I) direct estimation of FGM/C was performed through a nationwide survey of all midwifery practices in the Netherlands and (II) the extrapolation model was adopted for indirect estimation of FGM/C, by applying population-based-survey data on FGM/C in country of origin to migrant women who gave birth in 2018 in the Netherlands.

**Results:**

A nationwide survey among primary care midwifery practices that provided care for 57.5% of all deliveries in 2018 in the Netherlands, reported 523 cases of FGM/C, constituting FGM/C prevalence of 0.54%. The indirect estimation of FGM/C in an extrapolation-model resulted in an estimated prevalence of 1.55%. Possible reasons for the difference in FGM/C prevalence between direct- and indirect estimation include that the midwives were not being able to recognize, record or classify FGM/C, referral to an obstetrician before assessing FGM/C status of women and selective responding to the survey. Also, migrants might differ from people in their country of origin in terms of acculturation toward discontinuation of the practice. This may have contributed to the higher indirect-estimation of FGM/C compared to direct estimation of FGM/C.

**Conclusions:**

The current study has provided insight into direct estimation of FGM/C through a survey of midwifery practices in the Netherlands. Evidence based on midwifery practices data can be regarded as a minimum benchmark for actual prevalence among the subpopulation of women who gave birth in a given year.

## Background

Female Genital Mutilation or Cutting (FGM/C) refers to ‘all procedures involving the partial or total removal of the external female genitalia, or other injury to the female genital organs for non-medical reasons’ [1]. Four types of FGM/C are classified, ranging from clitoridectomy (the excision of the prepuce, with or without excision of part or all of the clitoris) to infibulation (the excision of part- or all of the external genitalia and stitching or narrowing of the vaginal opening) [1]. FGM/C has harmful health consequences [2–7], especially for women during pregnancy and childbirth [8, 9]. Evidence shows a significant higher prevalence of ‘prolonged labor, obstetric lacerations, instrumental delivery, obstetric haemorrhage and difficulty delivery’ among women with FGM/C compared to women without FGM/C [9]. Other obstetric consequences of FGM/C include fear of childbirth, difficulty in intrapartum monitoring, difficulty in catheterization during labor, wound infection and retention of lochia [8]. In addition to obstetric complications, infants born from women with FGM/C are at increased risk of stillbirth and early neonatal death [8].

FGM/C is predominantly practiced in countries in Africa, the Middle East and Asia, where it is estimated that over 200 million girls and women have undergone FGM/C [10]. With an increase in migration, FGM/C is now encountered in high-resource countries such as the Netherlands. It is estimated that about 41,000 girls and women are living in the Netherlands with effects of FGM/C, of whom 71% were of reproductive age [11]. The migration from countries where FGM/C is concentrated to non-practicing countries will even further increase [12, 13]. Consequently, in the near future, more and more health care providers are expected to provide care for women with FGM/C. Therefore, it is necessary to have insight into the prevalence of FGM/C, especially in the obstetric care setting.

Currently, the extrapolation-model has been widely used in high-resource countries to estimate the number of women with FGM/C [14]. The extrapolation-method, also known as ‘indirect estimation’, extrapolates information on FGM/C prevalence in countries of origin and applies country-prevalence (eventually with corrections) to female migrants in countries of destination. Although the extrapolation-model has certain advantages such as not being complex and having low costs, the model may not provide an accurate picture of the practice in diaspora [15], especially regarding mechanisms of migration flows selection that could lead to underestimation- or overestimation of the prevalence based on the country of origin data and changes in FGM/C practice after taking residence in country of destination that primarily affect young girls. Therefore, it is crucial to validate the robustness of indirect estimates of FGM/C through direct estimation of FGM/C.

In the Netherlands, primary-care midwives are responsible for care provision to women with low-risk of pregnancy complications. Women are referred to an obstetrician in case of complications during pregnancy or childbirth [16, 17]. Since 89% of pregnant women in the Netherlands visit a primary care midwifery practice at least once during their pregnancy [18], we choose to directly estimate the prevalence of FGM/C through a survey of midwifery practices. Pregnancy presents the midwives with a natural opportunity to examine female genitalia.

The aims of this study were (I) to estimate the prevalence of FGM/C through a nationwide survey among midwifery practices in the Netherlands, and (II) to compare this estimation with indirect estimation of FGM/C based on an extrapolation-model.

## Methods

### Direct estimation

#### Study population and design

A nationwide survey was conducted to determine the prevalence of FGM/C in primary-care midwifery practices in the Netherlands. In March 2019, a letter was sent to all these midwifery practices explaining the purpose of the study, including a questionnaire and a prepaid return envelope. The questionnaire was previewed and screened by the Royal Dutch Organization of Midwives (KNOV) and then improved before sent out. A reminder e-mail was sent 3 weeks later to practices that had not yet completed the paper questionnaire. They were given the option to complete a Web-based version of this questionnaire. Two weeks later, they were reminded by phone in case the questionnaire was not returned. In total, 503 midwifery practices were invited to participate in the survey; of these, 336 (66.8%) returned the questionnaires. Practices who did not return the questionnaire were mainly located in the largest cities (i.e., Amsterdam, Rotterdam, Den Haag). Respondents were eliminated if they did not fully answer the questionnaires, resulting in a working sample of 319 midwifery practices.

#### Measures

The questionnaire asked about (I) the number of women who received care in 2018 during pregnancy, birth giving and postpartum, (II) the number of women with FGM/C who received midwifery care in 2018, and (III) the number of women with infibulation (Type III). We did not include a question about the country of origin, because the country of origin is not routinely documented in medical records. Further, in order to reduce the possibility of recall error, respondents were asked to retrieve FGM/C cases from medical records. Since health care providers may experience difficulties in identifying and recording FGM/C [19–21], respondents were also asked to indicate if they were certain about the number of circumcised women reported. Finally, a free-text field was included at the end of the survey to allow participants to comment on their answers if necessary.

#### Data analysis

Prevalence of FGM/C and its confidence intervals (CIs) were calculated as described by Snedecor and Cochran [22]. Using the free-text field, some participants indicated not being able to record or retrieve FGM/C cases from medical records. Therefore, using the same procedure as described above, we analyzed whether the level of uncertainty about their assessment and data retrieval from medical records influenced the reported FGM/C prevalence per midwifery practice.

### Indirect estimation

#### Methodological approach and study population

We regarded the indirect estimation of FGM/C as a theoretical estimation of the expected FGM/C prevalence among women giving birth. For indirect estimation of FGM/C, we adopted the extrapolation-model and its underlying procedure, as described in detail in previous work [11]. In this study, we combined age-specific FGM/C prevalence in the country of origin with the age composition of first-generation migrant women who gave birth in the Netherlands. When necessary, the prevalence estimates were adjusted for variations in FGM/C prevalence across regions within the country of origin.

In this study, first-generation migrants are considered girls and women who migrated from one of the 29 countries with available nationally representative information on FGM/C, whereas second-generation migrants are considered girls born in the Netherlands to at least one parent who has migrated from one of these countries [11, 23].

#### Data sources and data analysis

Statistics Netherlands (CBS) provided microdata on first- and second-generation women who gave birth in 2018 in the Netherlands (*n* = 4598) by age, country of origin, birth place and date of arrival in the Netherlands. We excluded data from 546 first-generation women who missed information primarily regarding age and/or birth place. Unfortunately, data on ‘migration background’ of second-generation women (*n* = 236) were also missing, consequently they were excluded from the analysis. The final analysis was performed on 3816 first-generation women who gave birth in 2018 in the Netherlands.

There is a wide variation in the prevalence of FGM/C with both within and across countries where the practice is concentrated [24]. Therefore, adjustments were made to prevalence data to account for variation in FGM/C prevalence across regions in the country of origin. Therefore, we processed the dataset by regrouping migrant girls and women according to region within their countries of origin, using Microsoft Office Excel (2016) and IBM SPSS Statistics (version 25.0. Finally, data on the prevalence of FGM/C for countries of origin were extracted from the Demographic and Health Survey (DHS; www.dhsprogram.com) and the Multiple Indicator Cluster Survey (MICS; www.mics.unicef.org/surveys) country reports, which are publicly-accessible. DHS and MICS are nationally representative household surveys in low- and middle-income countries that collect information on a variety of indicators related to health, including FGM/C [24].

## Results

### Direct estimation

The total study population comprised 96,932 pregnant women, including women from non-FGM/C practicing countries, who had received care in 2018 from 319 midwifery practices in the Netherlands, comprising 57.5% of all deliveries in the Netherlands in 2018. A total of 168 out of 319 (53%) midwifery practices reported providing care to women with FGM/C. In these practices a total of 523 FGM/C cases were reported, resulting in a prevalence of 0.54% (95% Confidence Interval 0.536–0.543) in about 97,000 women receiving care during pregnancy, delivery and postpartum (see Table 1). About 32% of the 523 FGM/C cases were reported as infibulation (Type III).

**Table 1:** Estimated FGM/C prevalence in midwifery practices, according to certainty of recognition and retrieval medical records

	Number of midwifery practices	Number of women in care	Number of cut women in care	Prevalence (%)	95% Confidence Interval
All midwifery practices	319	96,932	523	0.54	0.536–0.543
Midwifery practices with cut women in care	167	60,651	522^a^	0.86	0.854–0.867
*Certain. retrieved*	25	26,040	198	0.76	0.751–0.770
*Certain. unretrieved*	74	6555	38	0.58	0.566–0.594
*Uncertain. retrieved*	21	9104	55	0.60	0.592–0.617
*Uncertain. unretrieved*	47	18,952	231	1.22	1.202–1.236

^a^Data on ‘*certainty’* and ‘*retrieval from medical records’* were missing from one midwifery practice

Using the free-text field, some participants indicated not being able to record or retrieve FGM/C cases from medical records. As shown in Table 1, FGM/C prevalence among midwifery practices who were sure of their assessment and who were able to retrieve FGM/C cases from records was 0.76% in contrast to 1.22% of practices who were uncertain and were not able to retrieve the numbers from medical records.

### Indirect estimation

Results from indirect estimation of FGM/C show that in 2018, 168,525 women gave birth, of whom 3816 were first-generation women whose country of origin is one of the 29 countries where FGM/C is concentrated (see Additional file 1). Of these women, about 2614 have most probably undergone FGM/C. Also, about 40% of these women are estimated to have been infibulated. Based on these figures, the expected FGM/C prevalence in women giving birth was thus estimated at 1.55%.

## Discussion

In this nationwide survey of primary-care midwifery practices on the prevalence of FGM/C, 336 of all eligible 503 practices participated, resulting in a 66.8% response, comprising 57.5% of all deliveries in 2018 in the Netherlands. Midwives reported 523 cases of FGM/C, constituting a prevalence of 0.54%. This indicates that 5 in 1000 women were reported as having undergone some form of FGM/C. This estimate was influenced by the extent to which midwives were certain about their assessment and their ability to retrieve FGM/C cases from medical records. The indirect estimation of FGM/C in an extrapolation-model resulted in an estimated prevalence of 1.55%.

Several reasons could explain the lower prevalence of FGM/C in midwifery practices as compared to indirect estimates of FGM/C. First, midwives reported on only 57.5% of the pregnant population in the Netherlands. Nonresponding midwives were mainly practicing in the larger urban areas (i.e., Amsterdam, Den Haag, Rotterdam). In the sensitivity analysis, we found no effect of urbanisation on the prevalence of FGM/C. Hence, the lower response in the larger cities in the Netherlands will most likely not have influenced our estimate of FGM/C prevalence. Given the lack of information regarding the countries of origin of women whose FGM/C status was provided by the midwifery practices, it was not possible to control for differences between the compositional differences between the population covered in the direct estimation exercise and data provided by the Statistics Netherlands. Second, recent studies show that health care providers experience difficulties recognizing, classifying and recording FGM/C due to, among others, demanding work schedules and insufficient awareness of FGM/C [19–21]. This corresponds to our findings of midwives not being able to record or retrieve FGM/C cases from medical records. Consequently, as we have demonstrated, not being able to retrieve FGM/C cases from records and uncertainty about FGM/C cases reported is associated with overestimating FGM/C cases. Therefore, in order to obtain accurate information about the occurrence of FGM/C, data regarding FGM/C should be systemically recorded in the national registration system. Finally, in the Netherlands, about 37.2% of pregnant women are referred to obstetricians during their pregnancy [18]. Hence, it is possible that FGM/C status of these women was not assessed by the midwives, who are the first point of contact in the Netherlands, before referral to an obstetrician, resulting in underreporting of FGM/C cases; this could be the case especially for cut women who may experience complications.

Using the extrapolation-model, it is also possible that we have overestimated the (expected) prevalence of FGM/C in women giving birth. Evidence shows that migrants represent a selected sample of people compared to stayers at the country of origin, that may originate from upper and middle-class families supporting the discontinuation of the practice [25]. There is evidence indicating that migration is a selective process [26]. Previous studies have documented that migrants are usually younger, wealthier and more educated than their counterparts in the country of origin. In many countries where FGM/C is practiced, lower age and higher levels of wealth and education or urban residence are often associated with lower occurrence of FGM/C. As a consequence, combining data on FGM/C prevalence in country of origin with data on female migrants in country of destination is likely to overestimate most country related indirect estimates of FGM/C prevalence. Although we were able to adjust for variation in the FGM/C prevalence among regions in countries of origin and age, a future study would need to adjust FGM/C prevalence according to other components of the selection hypothesis (i.e., educational level and wealth).

To our best knowledge, only the study by Korfker et al. [26] is the closest to ours. In their study on the prevalence of FGM/C among women delivering in the Netherlands, they reported direct- and indirect estimates of 0.32 and 0.7%, respectively. For indirect estimation of FGM/C, the authors have combined the number of women delivering from 15 countries where FGM/C is practiced with the national prevalence of FGM/C in their countries of origin. However, the authors were not able to correct indirect estimation on the basis of women’s birth places and ages upon arrival in the Netherlands, which may have resulted in under- or overestimation of FGM/C. In contrast, adjustments were made in the current study to account for variation in FGM/C prevalence among regions in the country of origin and age upon arrival in the Netherlands, possibly providing a more accurate prevalence estimate. Nonetheless, indirect estimation of FGM/C has methodological limitations, including possible inaccuracy of estimation, for instance, related to the process of social, geographical and selection of migrants [15]. Furthermore, in our survey about 32% of FGM/C cases were classified as infibulation, while in the study by Korfker et al. [26] slightly higher prevalence of infibulation was found (40%). No other studies were found in the literature, it is therefore difficult to compare our findings with studies in other settings or other population groups.

Direct estimation of FGM/C is regarded as the preferred approach to estimate the prevalence of FGM/C in diaspora [15]. The current study has provided insight into direct estimation of FGM/C through a survey of midwifery practices in the Netherlands. Evidence based on midwifery practices data can be regarded as a minimum benchmark for the actual prevalence among the subpopulation of women who gave birth in a given year. The shortcomings of our direct estimation are inherent to its retrospective design, and the possible inaccuracies of FGM/C cases reported due to recall error. Despite these limitations, the present study still provides robust evidence of the presence of women with FGM/C in the Netherlands.

## Conclusions

In the present study, we aimed to estimate the prevalence of FGM/C through a survey of midwifery practices in the Netherlands. Midwives reported 523 cases of FGM/C, constituting a prevalence of 0.54%. This suggests that 5 in 1000 women were reported as having undergone some form of FGM/C. Evidence based on midwifery practices data can be regarded as a minimum benchmark for actual prevalence among the subpopulation of women who gave birth in a given year. Our findings underline the importance of appropriate healthcare for those who have undergone FGM/C. Therefore, capacity building for healthcare professionals such as midwives and the implementation of guidelines on the management of FGM/C, which are currently being developed by the Dutch Society of Obstetrics and Gynaecology (NVOG), should be priorities in the Netherlands.

## Supplementary information

**Additional file 1.** Demographic Health Survey (DHS) and Multiple Indicator Cluster Survey (MICS) data: country of origin, source and year of publication, overall prevalence and the prevalence of Type III; and Statistics Netherlands dataset on first-generation women giving birth; and estimated numbers of cut women giving birth by Type III.

## Data Availability

The DHS and MICS data used in this paper are publicly available on the respective websites (www.dhsprogram.com; www.mics.unicef.org/surveys). Data on the number of women who gave birth in the Netherlands and data regarding the nationwide survey of midwifery practices on the prevalence of FGM/C cannot be shared because of privacy concerns and legal restrictions on data containing sensitive (health) information. Data are available from the Ethics Committee (contact via the Medical Ethics Committee Erasmus MC at + 31 107033625 or at metc@erasmusmc.nl) for researchers who meet the criteria for access to confidential data.

## Abbreviations

FGM/C: Female Genital Mutilation or Cutting
KNOV: Royal Dutch Organization of Midwives
CBS: Statistics Netherlands
DHS: Demographic and Health Survey
MICS: Multiple Indicator Cluster Survey
