# Supplementary material for: Estimates of female genital mutilation/cutting in the Netherlands: a comparison between a nationwide survey in midwifery practices and extrapolation-model
Source: BMC Public Health. 2020 Jun 29;20:1033. doi: 10.1186/s12889-020-09151-0 (PMC7325136; doi:10.1186/s12889-020-09151-0)
Supplement: Supplementary file 1 — Additional file 1. Demographic Health Survey (DHS) and Multiple Indicator Cluster Survey (MICS) data: country of origin, source and year of publication, overall prevalence and the prevalence of Type III; and Statistics Netherlands dataset on first-generation women giving birth; and estimated numbers of cut women giving birth by Type III. [file 12889_2020_9151_MOESM1_ESM.docx]

**Supplementary information**

Additional file 1 – Demographic Health Survey (DHS) and Multiple Indicator Cluster Survey (MICS) data: country of origin, source and year of publication, overall prevalence and the prevalence of Type III; and Statistics Netherlands dataset on first-generation women giving birth; and estimated numbers of cut women giving birth by Type III.

| **Country of origin** |  | **DHS and MICS-data** | | | | |  | **Statistics Netherlands** |  | **Undergone FGM/C** | |
| --- | --- | --- | --- | --- | --- | --- | --- | --- | --- | --- | --- |
|  |  | Source | Year of publication | Overall FGM/C prevalence (%) |  | Prevalence of Type III (%) |  | Number of women delivering |  | Estimated number of cut women delivering | Type III |
| Benin |  | MICS | 2014 | 9.2 |  | 10.1 |  | 7 |  | 0.96 | 0.10 |
| Burkina Faso |  | DHS | 2010 | 75.8 |  | 1.2 |  | 5 |  | 4.15 | 0.05 |
| Cameroon |  | DHS | 2004 | 1.5 |  | 5.0 |  | 41 |  | 0.77 | 0.04 |
| Central African Republic |  | MICS | 2010 | 24.3 |  | 7.0 |  | 0 |  | - | - |
| Chad |  | DHS | 2014-15 | 38.4 |  | 9.4 |  | 2 |  | 0.86 | 0.08 |
| Côte d'Ivoire |  | DHS | 2011-12 | 38.2 |  | 8.7 |  | 18 |  | 6.44 | 0.56 |
| Djibouti |  | MICS | 2006 | 93.2 |  | 67.2 |  | 7 |  | 6.58 | 4.42 |
| Egypt |  | DHS | 2015 | 87.2 |  | 0.7 |  | 139 |  | 128.12 | 0.90 |
| Eritrea |  | PHS | 2010 | 83.0 |  | 38.6 |  | 569 |  | 459.39 | 177.33 |
| Ethiopia |  | DHS | 2016 | 65.2 |  | 6.5 |  | 852 |  | 607.96 | 39.52 |
| Gambia |  | DHS | 2013 | 74.9 |  | 0.0 |  | 23 |  | 17.61 | 0.00 |
| Ghana |  | MICS | 2011 | 3.8 |  | 7.9 |  | 238 |  | 5.74 | 0.45 |
| Guinea |  | DHS | 2012 | 96.9 |  | 7.5 |  | 122 |  | 119.04 | 8.93 |
| Guinea-Bissau |  | MICS | 2014 | 44.9 |  | 6.0 |  | 8 |  | 3.77 | 0.23 |
| Iraq |  | MICS | 2011 | 8.1 |  | 0.0 |  | 322 |  | 71.36 | 0.00 |
| Kenya |  | DHS | 2014 | 21.0 |  | 9.3 |  | 45 |  | 16.96 | 1.58 |
| Liberia |  | DHS | 2013 | 55.5 |  | 0.0 |  | 17 |  | 8.60 | 0.00 |
| Mali |  | DHS | 2012-13 | 91.4 |  | 10.6 |  | 4 |  | 3.67 | 0.39 |
| Mauritania |  | MICS | 2015 | 66.6 |  | 4.5 |  | 2 |  | 1.16 | 0.05 |
| Niger |  | DHS | 2012 | 2.0 |  | 6.3 |  | 2 |  | 0.12 | 0.01 |
| Nigeria |  | DHS | 2013 | 24.8 |  | 5.3 |  | 143 |  | 50.37 | 2.67 |
| Senegal |  | DHS | 2016 | 22.7 |  | 7.1 |  | 15 |  | 3.74 | 0.27 |
| Sierra Leone |  | DHS | 2013 | 89.6 |  | 9.0 |  | 69 |  | 65.26 | 5.87 |
| Somalia |  | MICS | 2006 | 97.9 |  | 79.3 |  | 889 |  | 874.84 | 693.75 |
| Sudan |  | MICS | 2014 | 86.6 |  | 77.0 |  | 173 |  | 151.50 | 116.66 |
| United Republic of Tanzania |  | DHS | 2015-16 | 10.0 |  | 6.6 |  | 10 |  | 0.85 | 0.06 |
| Togo |  | DHS | 2013-14 | 4.7 |  | 15.4 |  | 17 |  | 0.16 | 0.03 |
| Uganda |  | DHS | 2016 | 0.3 |  | 0.0 |  | 59 |  | 0.99 | 0.00 |
| Yemen |  | DHS | 2013 | 18.5 |  | 0.0 |  | 18 |  | 2.70 | 0.00 |
| **Total** |  |  |  |  |  |  |  | **3816** |  | **2613.69** | **1053.91** |
